# Common Brain Networks Between Major Depressive-Disorder Diagnosis and Symptoms of Depression That Are Validated for Independent Cohorts

**DOI:** 10.3389/fpsyt.2021.667881

**Published:** 2021-06-10

**Authors:** Ayumu Yamashita, Yuki Sakai, Takashi Yamada, Noriaki Yahata, Akira Kunimatsu, Naohiro Okada, Takashi Itahashi, Ryuichiro Hashimoto, Hiroto Mizuta, Naho Ichikawa, Masahiro Takamura, Go Okada, Hirotaka Yamagata, Kenichiro Harada, Koji Matsuo, Saori C. Tanaka, Mitsuo Kawato, Kiyoto Kasai, Nobumasa Kato, Hidehiko Takahashi, Yasumasa Okamoto, Okito Yamashita, Hiroshi Imamizu

**Affiliations:** ^1^Brain Information Communication Research Laboratory Group, Advanced Telecommunications Research Institutes International, Kyoto, Japan; ^2^Medical Institute of Developmental Disabilities Research, Showa University, Tokyo, Japan; ^3^Department of Neuropsychiatry, Graduate School of Medicine, The University of Tokyo, Tokyo, Japan; ^4^Quantum Life Informatics Group, Institute for Quantum Life Science, National Institutes for Quantum and Radiological Science and Technology, Chiba, Japan; ^5^Department of Molecular Imaging and Theranostics, National Institute of Radiological Sciences, National Institutes for Quantum and Radiological Science and Technology, Chiba, Japan; ^6^Department of Radiology, The Institute of Medical Science The University of Tokyo (IMSUT) Hospital, Institute of Medical Science, The University of Tokyo, Tokyo, Japan; ^7^Department of Radiology, Graduate School of Medicine, The University of Tokyo, Tokyo, Japan; ^8^The International Research Center for Neurointelligence (WPI-IRCN) at the University of Tokyo Institutes for Advanced Study (UTIAS), Tokyo, Japan; ^9^Department of Language Sciences, Tokyo Metropolitan University, Tokyo, Japan; ^10^Department of Psychiatry, Kyoto University Graduate School of Medicine, Kyoto, Japan; ^11^Department of Psychiatry and Neurosciences, Hiroshima University, Hiroshima, Japan; ^12^Division of Neuropsychiatry, Department of Neuroscience, Yamaguchi University Graduate School of Medicine, Yamaguchi, Japan; ^13^Department of Psychiatry, Faculty of Medicine, Saitama Medical University, Saitama, Japan; ^14^Center for Advanced Intelligence Project, Institute of Physical and Chemical Research (RIKEN), Tokyo, Japan; ^15^Department of Psychiatry and Behavioral Sciences, Graduate School of Medical and Dental Sciences, Tokyo Medical and Dental University, Tokyo, Japan; ^16^Department of Psychology, Graduate School of Humanities and Sociology, The University of Tokyo, Tokyo, Japan

**Keywords:** resting-state functional magnetic resonance imaging, machine learning, resting-state functional connectivity, major depressive disorder, depression symptoms

## Abstract

Large-scale neuroimaging data acquired and shared by multiple institutions are essential to advance neuroscientific understanding of pathophysiological mechanisms in psychiatric disorders, such as major depressive disorder (MDD). About 75% of studies that have applied machine learning technique to neuroimaging have been based on diagnoses by clinicians. However, an increasing number of studies have highlighted the difficulty in finding a clear association between existing clinical diagnostic categories and neurobiological abnormalities. Here, using resting-state functional magnetic resonance imaging, we determined and validated resting-state functional connectivity related to depression symptoms that were thought to be directly related to neurobiological abnormalities. We then compared the resting-state functional connectivity related to depression symptoms with that related to depression diagnosis that we recently identified. In particular, for the discovery dataset with 477 participants from 4 imaging sites, we removed site differences using our recently developed harmonization method and developed a brain network prediction model of depression symptoms (Beck Depression Inventory-II [BDI] score). The prediction model significantly predicted BDI score for an independent validation dataset with 439 participants from 4 different imaging sites. Finally, we found 3 common functional connections between those related to depression symptoms and those related to MDD diagnosis. These findings contribute to a deeper understanding of the neural circuitry of depressive symptoms in MDD, a hetero-symptomatic population, revealing the neural basis of MDD.

## Introduction

Major depressive disorder (MDD) is diagnosed when depression symptoms persist for more than 2 weeks, and is the world's most serious psychiatric disorder in terms of its social repercussions (1, 2). A substantial body of evidence supports the existence of brain network alterations in MDD (3–6). Some studies have succeeded in predicting MDD diagnosis from the brain network by using resting-state functional magnetic resonance imaging (rs-fMRI) (7–13). Rs-fMRI is a measurement to quantify the functional connection (FC) of correlated, spontaneous, blood-oxygen-level-dependent (BOLD) signal fluctuations (14–16).

However, an increasing number of studies have highlighted the difficulty of finding a clear association between existing clinical diagnostic categories and neurobiological abnormalities (17–19). This process is difficult because diagnosis is based on a complex assemblage of information, such as symptoms, syndromes, and clinical experience. The high comorbidity of structural, functional, and genetic abnormalities across psychiatric disorders exacerbates this difficulty (20–23). Therefore, the necessity of a symptom-based approach that directly investigates neurobiological abnormalities related to symptoms has been increasingly recognized (24–26). However, 75% of the 475 translational neuroimaging studies that used machine learning techniques focused only on “diagnoses,” classifying patients from controls, and only 2.8% focused on “symptoms” to predict continuous symptom scores (27). Thus, it is important to investigate FCs that are associated with depressed symptoms. Comparing them with FCs related to MDD diagnosis strengthens neuroscientific understanding, and aids future diagnosis and treatment of MDD. For instance, targeting FCs that are important for both diagnosis and symptoms may lead to more effective treatment that improves both diagnosis and symptoms. Another problem is that consistent conclusions regarding neurobiological abnormalities across studies could not be achieved, mainly because of large imaging-site differences in rs-fMRI data and overfitting to noise in discovery cohorts (28, 29). Accordingly, it is urgent to improve reliability of neuroimaging analysis for psychiatry using independent validation datasets from multiple imaging sites, which are different from imaging sites for discovery cohort (30–34).

In the present study, we determined and validated resting-state FCs related to depression symptoms in a data-driven, unbiased manner, using several imaging sites. We used the same population as that in our previous study that identified FCs related to MDD diagnosis (13). We compared FCs related to depression symptoms, which were newly identified in the current study, with those related to MDD diagnosis in the same population. We considered and satisfied 3 issues and conditions to ensure generalization of our network models of depressive symptoms in the independent validation dataset, which does not include imaging sites of the discovery dataset. First, to remove site differences in FCs, we used a novel traveling subject harmonization method. Second, we validated our network markers using a large, independent cohort collected from multiple imaging sites. Third, we avoided overfitting noise in the discovery dataset by using a sparse machine learning algorithm with the least absolute shrinkage and selection operator (LASSO) (35). We used LASSO because it allows us to select important feature simultaneously. As a result, to the best of our knowledge, we developed the first generalizable brain network prediction model for depression symptoms and found three common FCs between depression symptoms and MDD diagnosis.

## Results

### Datasets

Two rs-fMRI datasets were used for analyses. The “discovery dataset” included 713 participants (564 HCs from 4 sites, 149 patients with MDD from 3 sites; Table 1), and the “independent validation dataset” included 449 participants (264 HCs from independent 4 sites, 185 patients with MDD from independent 4 sites; Table 1). Data can be downloaded publicly from the DecNef Project Brain Data Repository (https://bicr-resource.atr.jp/srpbsopen/ and https://bicr.atr.jp/dcn/en/download/harmonization/). Imaging protocols and data availability from each site are described in Supplementary Table 1. We evaluated depression symptoms using the Beck Depression Inventory-II (BDI-II) score obtained from most participants in each dataset. To construct the BDI prediction model, we used participants in the discovery dataset with BDI-II score. We then validated our BDI prediction model using the independent validation dataset collected from multiple imaging sites. In total, we used 477 participants (367 HCs and 110 MDDs) in the discovery dataset and 439 patients (259 HCs and 180 MDDs) in the validation dataset. Clinical details such as medication information and comorbidities in patients with MDD are described in Supplementary Table 2.

**Table 1:** Demographic characteristics of participants in both datasets.

**Site**	**HC**				**MDD**				**ALL**			
	**Number**	**Male/female**	**Age (y)**	**BDI**	**Number**	**Male/female**	**Age (y)**	**BDI**	**Number**	**Male/female**	**Age (y)**	**BDI**
**Discover dataset**												
Center of Innovation in Hiroshima University (COI)	124 (123)	46/78	51.9 ± 13.4	8.2 ± 6.3	70 (70)	31/39	45.0 ± 12.5	26.2 ± 9.9	194 (193)	77/117	49.4 ± 13.5	14.7 ± 11.7
Kyoto University (KUT)	169 (139)	100/69	35.9 ± 13.6	6.0 ± 5.4	17 (17)	11/6	43.9 ± 13.3	27.7 ± 10.1	186 (156)	111/75	36.7 ± 13.7	8.3 ± 9.1
Showa University (SWA)	101 (97)	86/15	28.4 ± 7.9	4.4 ± 3.8	0	–	–	–	101 (97)	86/15	28.4 ± 7.9	4.4 ± 3.8
University of Tokyo (UTO)	170 (24)	78/92	35.6 ± 17.5	6.7 ± 6.5	62 (32)	36/26	38.7± 11.6	20.4 ± 11.4	232 (56)	114/118	36.4 ± 16.2	14.5 ± 11.8
Summary	564 (383)	310/254	38.0 ± 16.1	6.3 ± 5.6	149 (119)	78/71	42.3 ± 12.5	24.9 ± 10.7	713 (502)	388/325	38.9 ±15.5	10.7 ± 10.6
**Independent validation dataset**												
Hiroshima Kajikawa Hospital (HKH)	29 (29)	12/17	45.4 ± 9.5	5.1 ± 4.6	33 (33)	20/13	44.8 ± 11.5	28.5 ± 8.7	62 (62)	32/30	45.1 ± 10.5	17.6 ± 13.7
Hiroshima Rehabilitation Center (HRC)	49 (49)	13/36	41.7 ± 11.7	9.1 ± 8.5	16 (16)	6/10	40.5 ± 11.5	35.3 ± 9.5	65 (65)	19/46	41.4 ± 11.5	15.6 ± 14.3
Hiroshima University Hospital (HUH)	66 (66)	29/37	34.6 ± 13.0	6.9 ± 5.9	57 (57)	32/25	43.3 ± 12.2	30.9 ± 9.0	123 (123)	61/62	38.6 ± 13.3	18.0 ± 14.1
Yamaguchi University (UYA)	120 (120)	50/70	45.9 ± 19.5	7.1 ± 5.6	79 (78)	36/43	50.3 ± 13.6	29.7 ± 10.7	199 (198)	86/113	47.6 ± 17.5	16.0 ± 13.6
Summary	264 (264)	104/160	42.2 ± 16.5	7.2 ± 6.3	185 (184)	94/91	46.3 ± 13.0	30.3 ± 9.9	449 (448)	198/251	43.9 ± 15.3	16.7 ± 13.9

*Numbers in parentheses indicate numbers of participants with BDI score. All demographic distributions are matched between the MDD and HC populations in the discovery dataset (P > 0.05) except for BDI. The demographic distribution of age is matched between MDD and HC populations in the independent validation dataset (P > 0.05). Demographic distributions of the sex ratio and BDI were not matched between the MDD and HC populations in the validation dataset (P < 0.05). BDI, Beck Depression Inventory-II; HC, Healthy Control; MDD, Major Depressive Disorder*.

### Site-Difference Control in FC

Conventional preprocessing was performed (see Materials and Methods), and FC was defined based on a functional brain atlas consisting of 379 nodes (regions) covering the whole brain (36). Fisher's *z*-transformed Pearson correlation coefficients between preprocessed BOLD signal time courses of each possible pair of nodes were calculated and used to construct 379 × 379 symmetrical connectivity matrices in which each element represents a connection strength, or edge, between two nodes. We used 71,631 connectivity values (379 × 378/2) of the lower triangular matrix of the connectivity matrix.

To control for site differences in the FC, we applied a traveling subject harmonization method that removes site differences in FC (29). According to our previous study (29), differences in resting state FCs consist of measurement bias due to differences in fMRI protocols and MR scanners, and sampling bias due to recruitment of different participant populations. The magnitude of measurement bias was larger than the effects of disorders, whereas the magnitude of the sampling bias was comparable to the effects of disorders (29). Therefore, a reduction in site differences in FC is essential for generalization of network models in the validation dataset. In this method, measurement bias was estimated by fitting the regression model to FC values of all participants from the discovery dataset and the traveling subject dataset, wherein multiple participants travel to multiple imaging sites (see *Site difference control in FC* in Methods section). We can then subtract only the measurement bias while leaving important information due to differences in subjects among imaging sites. We applied the ComBat harmonization method (37–40) to control for site differences in the FC of the validation dataset because we did not have a traveling subject dataset for those sites.

### Reproducible FCs Related to Depression Symptoms and Shared Information With MDD Diagnosis

**Figure 1:**
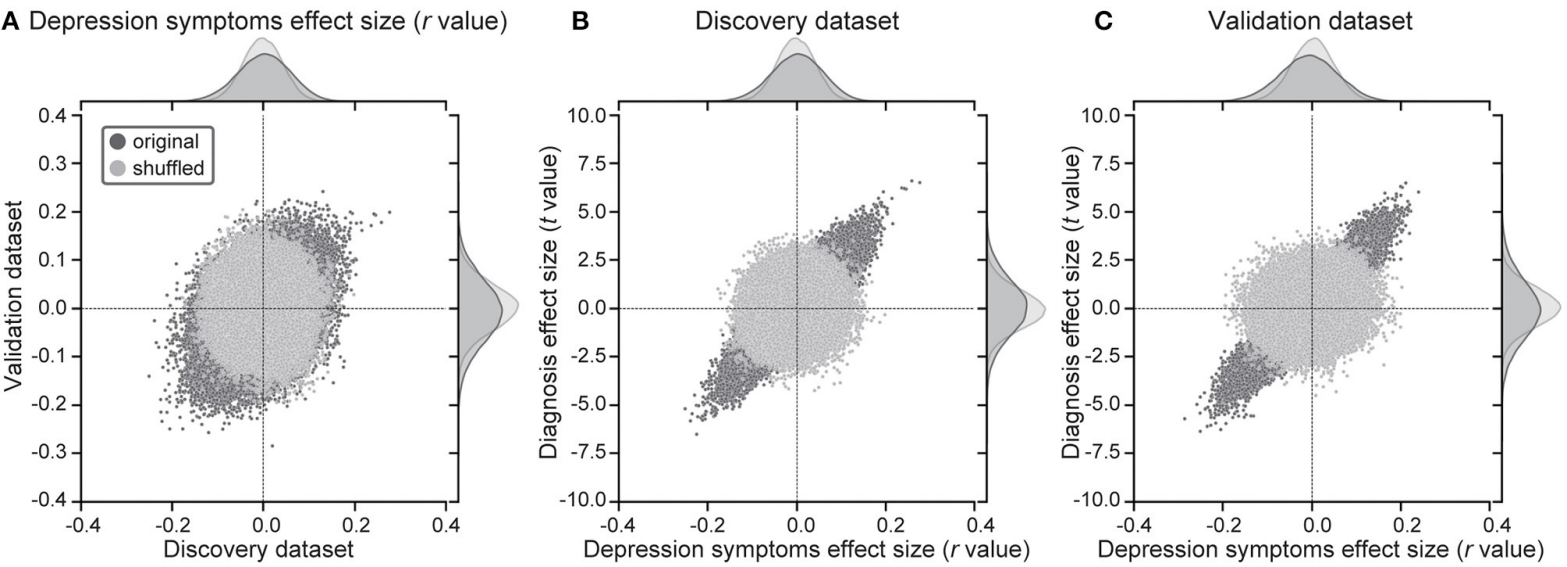
Results of mass univariate analysis. **(A)** Reproducibility across the 2 datasets regarding symptom effects. Scatter plots and histograms of depression symptom effect sizes (Pearson's correlation between BDI-II and functional connectivity strength: *r*-value). Each point in the scatter plots represents the symptom effect in the discovery dataset in the abscissa and that for the validation dataset in the ordinate for each functional connectivity. Original data are in black, while shuffled data in which subject information was permuted are in gray. **(B,C)** Shared information between diagnosis and symptom effects in both datasets. Scatter plots and histograms of diagnosis effect sizes (the difference in mean functional connectivity strengths between patients with depression and healthy groups: *t*-value) in the ordinate and depression symptom effect sizes (*r*-value) in the abscissa for all functional connectivity in the discovery dataset **(B)** and the validation dataset **(C)**. Original data are in black, while shuffled data in which subject information was permuted are in gray.

Utilizing a simple mass univariate analysis, we estimated the reproducibility of effect sizes by depression symptoms on individual FCs across the discovery and validation datasets. For the effect of a depressed symptom on each FC, we calculated the Pearson's correlation coefficient (*r*-value) between FC strength and BDI scores across all participants for every FC. A scatter plot (Figure 1A) shows the effect size for the discovery dataset in the abscissa and that for the validation dataset in the ordinate for each FC. We compared distributions of statistics in the discovery dataset to distributions in the shuffled data in which symptom severity was permuted across subjects. We found larger effects of symptoms in the original data in comparison to the shuffled data (Figure 1A, upper histogram). We confirmed that results were similar in the validation dataset (Figure 1A, right histogram). These results indicate that resting-state FCs contain consistent information across both datasets, regarding depression symptoms. To statistically evaluate the reproducibility of the effect on FCs, we calculated Spearman's correlation between the discovery and validation datasets regarding the above statistic (*r*-value). We found significant correlations between the two datasets (Spearman's *r*-value: *r*_(71629)_ = 0.367, 95% CI = [0.360 0.373], *R*^2^ = 0.13, [permutation test, *P* < 0.001, one-sided], Figure 1A). This result indicates that the effect of symptom severity was reproducible, even in the independent dataset acquired from completely different sites.

### Shared Information on FCs Between MDD Diagnosis and Depression Symptoms

We investigated whether FCs related to MDD diagnosis and FCs related to depression symptoms share information. For the effect of the diagnosis on each FC, we calculated the difference in the FC value across participants between HCs and MDDs (*t*-value). To this end, we calculated Spearman's correlation between *t*-values and *r*-values on FCs in the same dataset. We found high correlations (Discovery dataset: Spearman's *r*_(71629)_ = 0.848, 95% CI = [0.846 0.850], *R*^2^ = 0.72; Figure 1B, Independent validation dataset: *r*_(71629)_ = 0.903, 95% CI = [0.901 0.904], *R*^2^ = 0.81; Figure 1C). This indicates that shared information exists in FCs between MDD diagnosis and depression symptoms.

### Brain Network Prediction Model of Depression Symptoms Generalized to Completely Different Multisite Data

**Figure 2:**
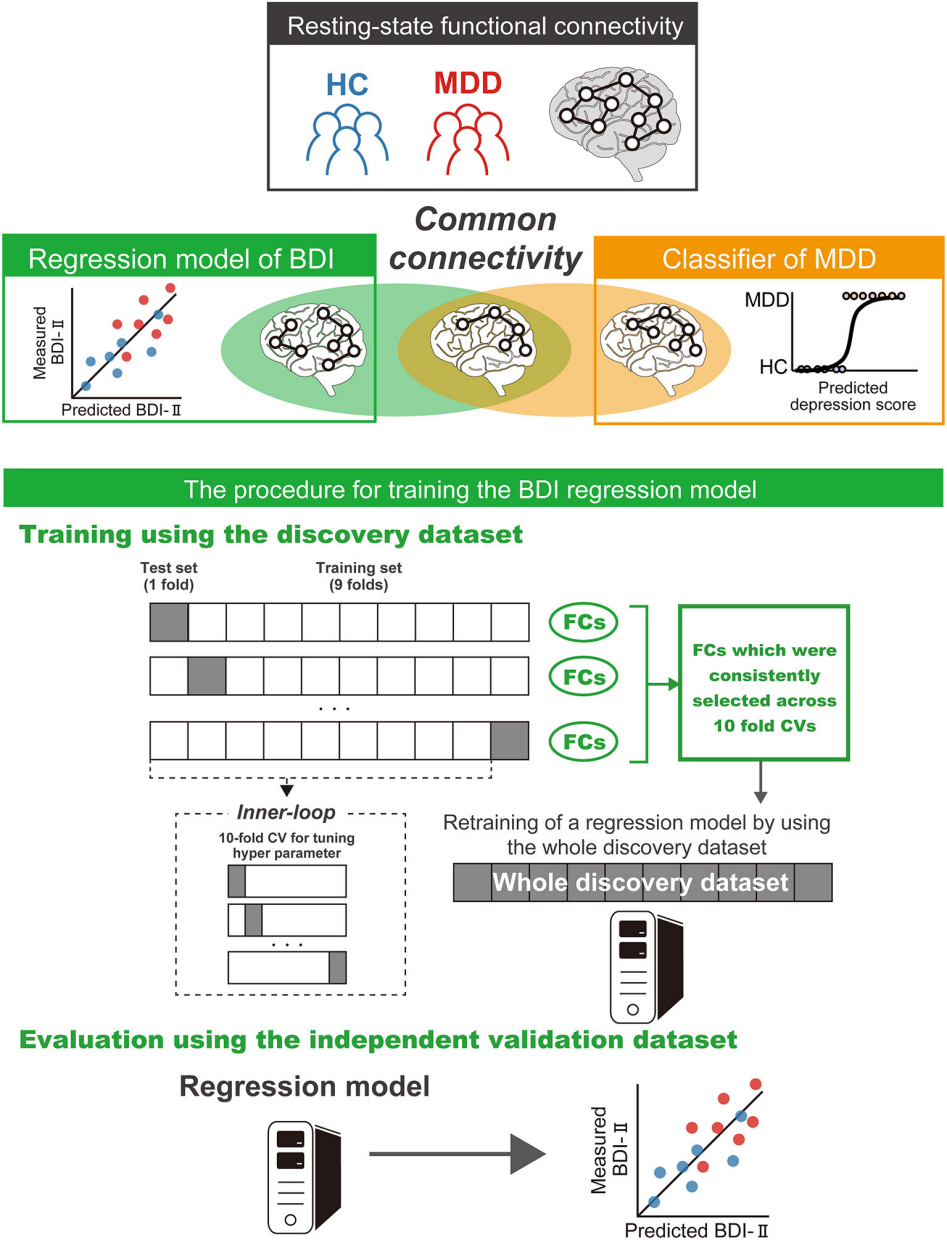
Schematic representation of the procedure for training the brain network prediction model and evaluation of its predictive power. The BDI regression model was constructed using the union of FC values selected by the embedded method in the discovery dataset. Generalization performance was evaluated by applying the constructed regression model to the independent validation dataset. The machine learning regression model is represented by PC cartoons. BDI, Beck Depression Inventory-II; CV, cross validation; MDD, major depressive disorder; HC, healthy control; FC, functional connectivity.

**Figure 3:**
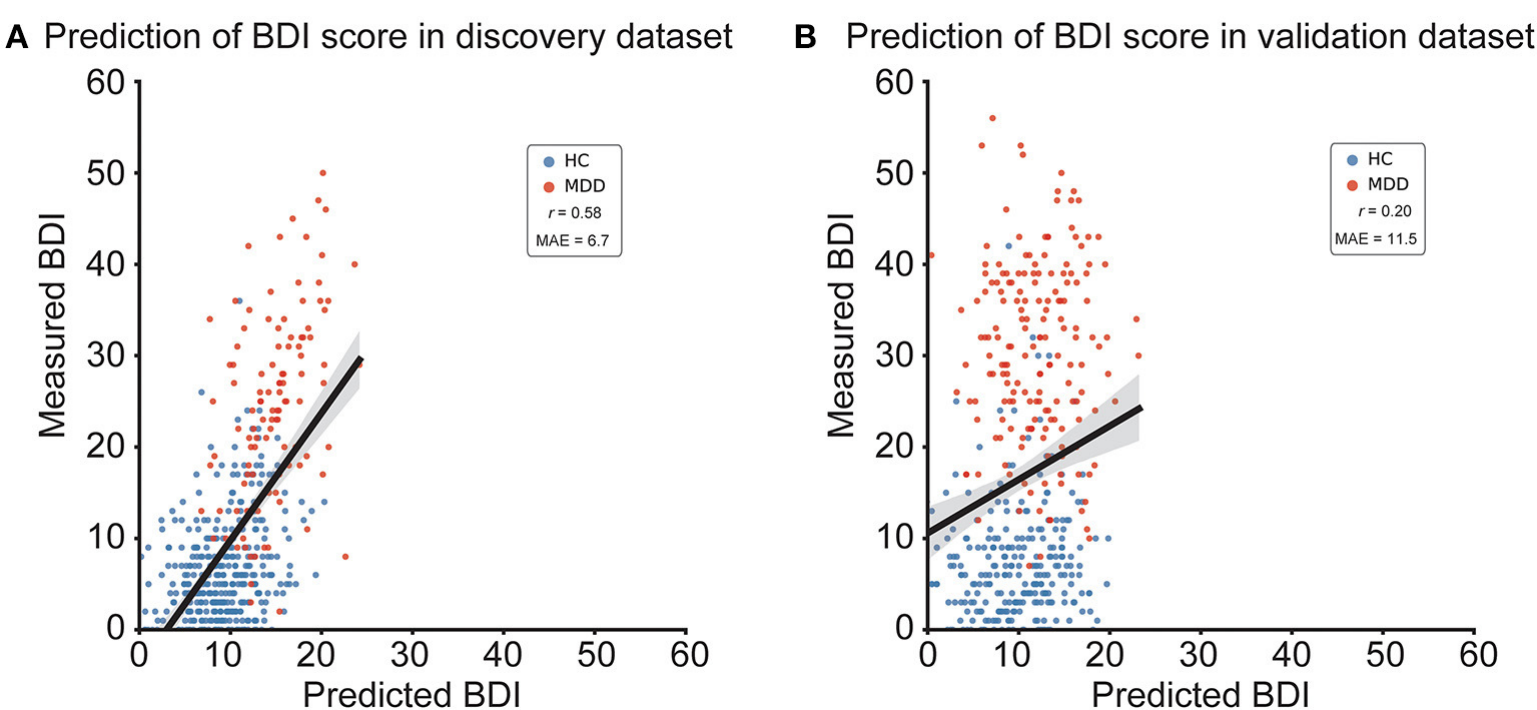
BDI regression model performances in the discovery and validation datasets. **(A)** Scatter plots of measured and predicted BDI in the discovery dataset. **(B)** Scatter plots of measured and predicted BDI in the independent validation dataset. The solid line indicates the linear regression of the measured BDI from the predicted BDI. The correlation coefficient (*r*) and mean absolute error (MAE) are shown. Each data point represents one participant. BDI, Beck Depression Inventory-II; HC, healthy control; MDD, major depressive disorder.

We constructed a brain network prediction model of the BDI score using the discovery dataset comprising 71,631 FC values. Based on our previous studies (12, 41–43), we assumed that depression symptoms were not associated with whole brain connectivity, but rather with a specific subset of connections. Furthermore, the number of FCs is 71,631, while the number of participants is about 500; thus, machine-learning algorithms can easily overfit the discovery cohort, inflating prediction performance, unless precautions are taken. Therefore, we used linear regression with the least absolute shrinkage and selection operator (LASSO), a sparse machine learning algorithm, to select the optimal subset of FCs (35, 44). We have already succeeded in constructing generalizable brain network markers of ASD, MDD, melancholic MDD, SCZ and obsessive compulsive disorder (12, 13, 41–43), using a similar sparse estimation method that automatically selects the most important connections. The prediction model was constructed based on the feature-selection procedure in which the embedded method with LASSO was performed (Figure 2) (45). We conducted a 10-fold nested CV procedure while optimizing hyperparameter in LASSO. We then constructed a regression model using the combination of FC values selected in all 10 folds in the training dataset (Figure 2). Based on our previous findings that final weights determined using the whole discovery dataset achieved better generalization performance (41), we determined final weights using the whole discovery dataset. Since this could have caused information leakage across folds for the evaluation in the discovery dataset, it was very important to confirm generalization performance by applying this regression model to an independent validation dataset, as described below. Finally, we calculated the mean absolute error (MAE) and Pearson's correlation coefficients between the predicted and measured BDI scores. The BDI score was well-predicted with a significant correlation (*r*_(475)_ = 0.58, 95% CI = [0.519 0.638], *R*^2^ = 0.34, *P* = 1.4 × 10^−44^, one-sided; MAE = 6.7; Figure 3A). Furthermore, a significant correlation was achieved for HC and MDD populations separately (HC, *r*_(365)_ = 0.28, 95% CI = [0.181 0.370], *R*^2^ = 0.08, *P* = 6.3 × 10^−8^, one-sided; MDD, *r*_(108)_ = 0.42, 95% CI = [0.258 0.567], *R*^2^ = 0.18, *P* = 3.7 × 10^−7^). Once again, cautiously, these results could have represented overfitted inflation because evaluation data are not independent. Therefore, correct assessment had to be based on results from the following independent validation dataset.

We tested the generalizability of the regression model using the independent validation dataset. We applied the trained regression model to the independent validation dataset and considered its output as the predicted BDI score. The BDI score was moderately well-predicted, with a significant correlation in the independent validation dataset (*r*_(437)_ = 0.20, 95% CI = [0.104 0.284], *R*^2^ = 0.038; MAE = 11.5; Figure 3B; permutation test, *P* < 0.01, one-sided). To test the statistical significance of BDI regression model performance, we performed a permutation test. We permuted the BDI scores of the discovery dataset, constructed the BDI regression model in the same way, and repeated this permutation procedure 100 times.

To rule out the possibility that the BDI regression model's performance was driven by confounds, we assessed whether we could predict output of the model (predicted BDI score) using age, sex, the amount of motion (frame-wise displacement [FD]) or a combination of head movement parameters (x, y, z, yaw, pitch, and roll), respectively, for the discovery dataset. As a result, we confirmed that these regressors did not predict output of the model based on age (*r*_(475)_ = −0.14, *p* = 0.0016), sex (*r*_(475)_ = 0.06, *p* = 0.18), the FD (*r*_(475)_ = −0.07, *p* = 0.098) or the combination of head movement parameters (*r*_(475)_ = 0.08, *p* = 0.061). These results indicated that the regression model's performance was unlikely to have been driven by confounds.

We further assessed prediction performance when we used a parcellation scheme other than Glasser's region of interest (ROI). We found that there was no large difference in prediction performance, due to ROI numbers or parcellation schemes (Supplementary Text 1).

### Important FCs for the Brain Network Markers

We examined important resting state FCs for depression symptoms by extracting important FCs related to the BDI regression model. We counted the number of times an FC was selected during the 10-fold CV. We permuted the BDI scores of the discovery dataset, conducted a 10-fold CV, and repeated this permutation procedure 100 times. We then identified the FCs that were important if the number was significantly higher than the threshold for randomness, according to a permutation test. We used the number of counts for each FC selected by the LASSO during 10-fold CV as a statistic in every permutation dataset. To avoid the multiple comparison problem, we set a null distribution as the distribution of the maximum counts over all FCs and set statistical significance to a certain threshold (permutation test, *P* < 0.05, one-sided). Finally, to make it clear that the selected FCs were not affected by confounders, we introduced confounding factors (age, sex, FD, and 6 motion parameters) to the final model that used FCs selected by the LASSO during 10-fold CV. We determined the weights applying the LASSO to the whole discovery dataset, and if selected FCs were still selected by the LASSO, it indicated that these FCs were not affected by confounders.

**Figure 4:**
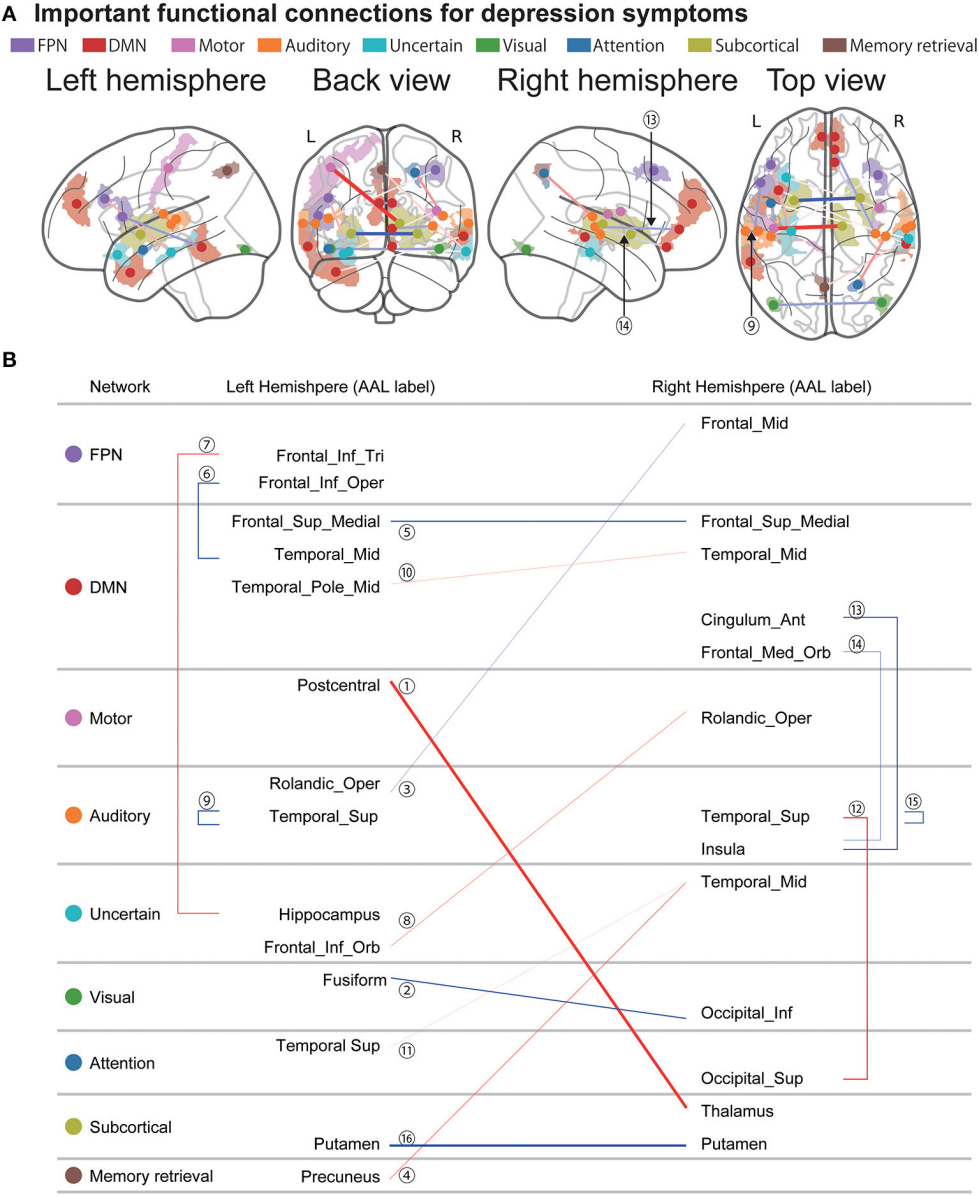
Important FCs for depression symptoms. **(A)** The 16 functional connections (FCs) viewed from left, back, right, and top. Interhemispheric connections are shown in the back and top views only. Regions are color-coded according to the intrinsic network. The state of functional connectivity exhibits characteristics of the correlation with depression symptoms as follows. Thinner and thicker connections indicate weaker and stronger correlations with depression symptoms in the validation dataset. Blue and red connections indicate negative and positive correlations, respectively. **(B)** Listed here are the laterality and anatomical identification of the ROI, as identified by Anatomical Automatic Labeling (AAL) and associated intrinsic networks related to the 17 FCs. MDD, major depressive disorder; DMN, default mode network; FPN, fronto-parietal network.

**Figure 5:**
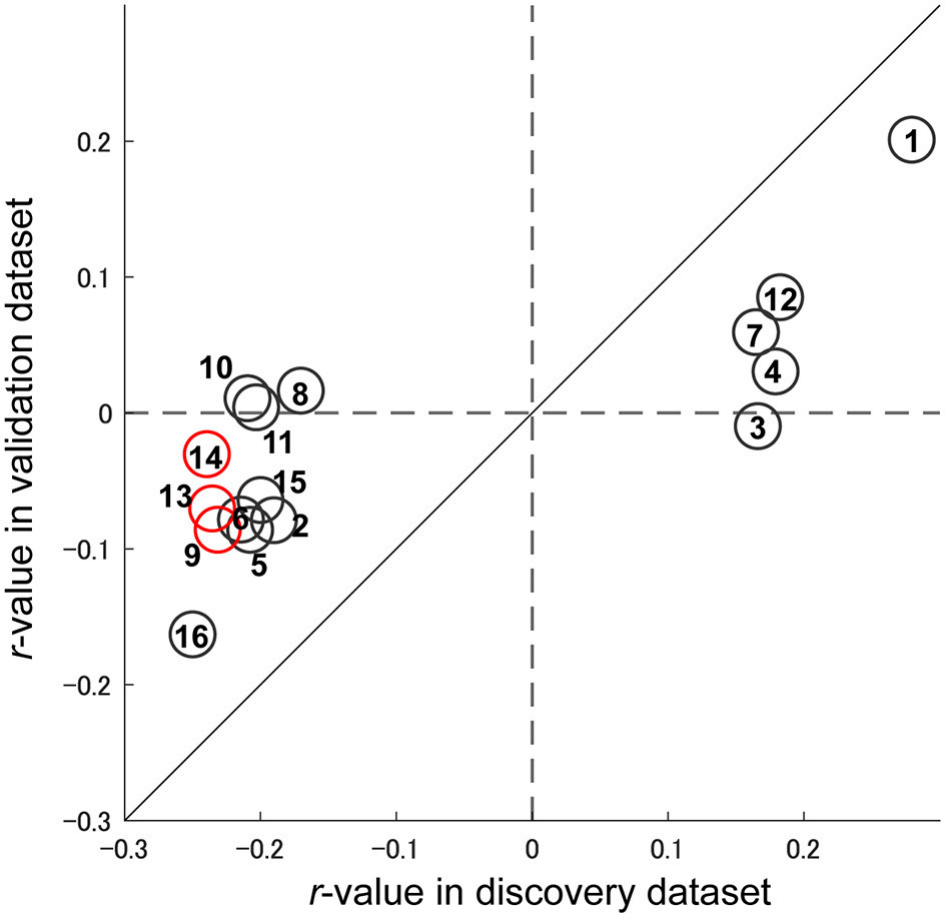
Reproducibility of important FCs regarding symptom effects. Scatter plot of the depression symptom effect size (Pearson's correlation between BDI-II and FC strength: *r*-value). Each circle represents the symptom effect in the discovery dataset in the abscissa and that for the validation dataset in the ordinate for each FC. Red circles indicate common FCs between the major depressive disorder diagnosis and depression symptoms models. The number in the circle is the number of the FC, as in Figure 4 and Supplementary Table 3.

Figure 4A shows the spatial distribution of the 16 FCs related to depression symptoms that machine learning algorithms automatically identified in the data without bias. We already identified 25 FCs related to the MDD diagnosis in our previous study (13) (Supplementary Text 2 and Supplementary Figure 1). We then compared these FCs. As a result, we found that three FCs were common between the diagnosis and symptom models. We hereafter summarize characteristics of these FCs. First, these connections were connections within left superior temporal gyrus (FC#9) between the right insula and the right frontal medial orbital cortex (FC#13), and between the right insula and the right cingulum anterior cortex (FC#14) (Figure 4B). These FCs revealed a negative correlation with BDI score (Figure 5). Two of three FCs (FC#13, and #14) were related to insula (Figure 4B). Second, one of the three common FCs (FC#14) was related to the subgenual anterior cingulate cortex (sgACC) (Figure 4B). Third, the FC between the left postcentral and right thalamus revealed the largest positive correlation with BDI score (FC#1) in the validation dataset (Figure 5). Fourth, 7 of the 16 FCs were related to the temporal lobes. A detailed list of the FCs is provided in Supplementary Table 3, and details of these FCs are examined in the Discussion section.

## Discussion

In this study, we considered conditions and resolved difficulties to ensure the generalizability of our brain network prediction model of depression symptoms in the validation dataset, which did not include any imaging sites from the discovery dataset. We succeeded in generalizing our prediction model to the large, independent validation dataset. This generalization ensures scientific reproducibility and the clinical applicability of rs-fMRI. We believe that the current study has two important significances. First, our prediction model is based on objective, biological biomarkers of resting-state functional connectivity compared to BDI measurements. To date, the lack of biological, objective biomarkers for severity of MDD is a major problem in psychiatry for drug discovery, treatment selection, and so on. Second, we found important functional connections involved in depression symptoms. The discovery of brain connectivity related to depression symptoms is expected to lead to novel treatment for MDD, such as neurofeedback and transcranial magnetic stimulation, by targeting functional connections.

Machine learning algorithms reliably identified 16 FCs that are important for depression symptoms and 3 of them were also important FCs for MDD diagnosis (13). These 3 connections include the connection within the left superior temporal gyrus (FC#9) between the right insula and right frontal medial orbital cortex (FC#13), and between the right insula and the right cingulum anterior cortex (FC#14).

These FCs have the following characteristics:

(1) They revealed the negative correlation with BDI score.

(2) Two of 3FCs (FC#13, and #14) were related to insula. Abnormalities in the insula are not only found in patients with MDD (46, 47), but are also reported as common abnormalities (reduced gray matter volume) among psychiatric disorders (22). Therefore, connectivity associated with the insula is a potential neurobiological dimension to understand a multi-spectrum disorder.

(3) One of the 3 shared FCs (FC#14) was related to the sgACC. According to a previous study, the sgACC is metabolically overactive in treatment-resistant depression and is an important treatment target of deep brain stimulation for MDD (48).

(4) The FC between the left postcentral and right thalamus revealed the largest positive correlation with BDI score (FC#1) in the validation dataset. A previous study showed that compared with HCs, patients with MDD have enhanced functional connectivity between the thalamus and somatosensory cortex (49). Furthermore, FC strength is negatively correlated with the Snaith-Hamilton Pleasure Scale (SHAPS), which assesses affective experience (50), neuropsychological function and continuous attention level (49).

(5) Seven of the 17 FCs (FC#4, #6, #9, #10, #11, #12, and #15) were related to temporal cortical regions. According to a previous study that used over 10,000 samples from 20 sites, patients with MDD have thinner cortical gray matter than HCs in the temporal lobes, orbitofrontal cortex, anterior and posterior cingulate, and insula (51). These brain regions are highly consistent with brain regions found in this study and may represent changes in cortical gray matter. Furthermore, these brain regions are related to biotypes of MDD implicating clinical features of negative bias, anxious avoidance, threat dysregulation, inattention, and cognitive dyscontrol, respectively (52). These results indicate that we need further analyses to clarify how the regression model's output and abnormalities in each FC are associated with cognitive and affective functions.

While research based on diagnosis of single disorders has proven fruitful in the field of psychiatric disorders, it has been more important to conduct dimensional and transdiagnostic research. Recently, the Research Domain Criteria (RDoC) initiative seeks to redefine and identify subtypes of psychiatric disorders in terms of biological systems, without relying on diagnoses based solely on symptoms and signs (18). This initiative is expected to inform our understanding of heterogeneous and overlapping clinical presentations of psychotic disorders. In particular, we believe that dimensional, transdiagnostic, longitudinal, and therapeutic approaches are driving the field toward more precise biomarkers in mental health.

As with dimensional and transdiagnostic approaches, even though our current study focuses on depression symptoms, we were able to identify brain-based dimensions of psychopathology such as mood, psychosis, fear and so on. Connectivity-guided dimensions of psychopathology that cross clinical diagnostic categories have been delineated (25), and in the future we should be able to redefine psychiatric disorders in a unified manner, based on biological brain networks by locating patients with multiple psychiatric disorders in these dimensions.

In this study, we predicted participants' current depression symptoms. However, it is also important to investigate from a longitudinal point of view. For example, in the future, we may be able to investigate the effect of treatments such as drugs, by comparing pre- and post-outputs of brain network prediction models or FCs that are important for symptoms. Antidepressants have a heterogeneous effect on functional connectivity underlying melancholic depression (12), so it may become possible to quantitatively evaluate effects of drugs and other treatments based on brain network markers.

Finally, identification of biomarkers that determine therapeutic targets, such as theranostic biomarkers (53, 54), could allow more personalized treatment approaches. The 16 FCs discovered in this study are promising candidates as theranostic biomarkers for depression symptoms. Future work should investigate whether modulation of FCs could be an effective treatment for depression symptoms by using an intervention method with regard to FCs, such as functional connectivity neurofeedback training (53–59).

## Materials and Methods

### Ethics Statement

All participants provided written informed consent. All recruitment procedures and experimental protocols were approved by the institutional review boards of our respective institutions (Advanced Telecommunications Research Institute International [approval numbers: 13–133, 14–133, 15–133, 16–133, 17–133, and 18–133], Hiroshima University [E-38], Kyoto Prefectural University of Medicine [RBMR-C-1098], Showa University [SWA] [B-2014-019 and UMIN000016134], the University of Tokyo [UTO] Faculty of Medicine [3150], Kyoto University [C809 and R0027], and Yamaguchi University [H23-153 and H25-85]) and were conducted in accordance with the Declaration of Helsinki.

### Participants

Two rs-fMRI datasets were used for our analyses: (1) The discovery dataset included 713 participants (564 HCs from 4 sites, 149 MDD patients from 3 sites; Table 1). Each participant underwent a single rs-fMRI session, which lasted 10 min. Within the Japanese SRPBS DecNef project, we planned to acquire the rs-fMRI data using a unified imaging protocol (Supplementary Table 1; http://bicr.atr.jp/rs-fmri-protocol-2/). However, there were 2 erroneous phase-encoding directions (P → A and A → P). In addition, different sites had different MRI hardware (Supplementary Table 1). During the rs-fMRI scans, participants were instructed to “Relax. Stay Awake. Fixate on the central crosshair mark, and do not concentrate on specific things.” (2) The independent validation dataset included 449 participants (264 HCs from independent 4 sites, 185 MDD patients from independent 4 sites; Table 1). We acquired the data following protocols reported in Supplementary Table 1. Imaging sites were different from those in the discovery dataset. Each participant underwent a single rs-fMRI session lasting 5 or 8 min. We acquired this dataset in other projects since 2008, rather than as part of the SRPBS DecNef. Datasets from Hiroshima University Hospital (HUH), Hiroshima Kajikawa Hospital (HKH), and Hiroshima Rehabilitation Center (HRC), as part of the independent validation dataset, were acquired by “Development of diagnosis and treatment techniques for patients with severe intractable depression and insensitivity to antidepressant treatments, based on molecular and cellular studies on BDNF and depression” of the Japan Science and Technology Agency Core Research for Evolutional Science and Technology (CREST) since 2008 and by “Understanding the neurocircuit—molecular mechanism underlying pathophysiology of depression and the development of its neuroscience-based diagnosis and treatment” of the SRPBS since 2011. Dataset from Yamaguchi University (UYA) was acquired by “Exploration of the biological markers for discrimination of heterogeneous pathophysiology of major depressive disorder” of the SRPBS since 2012. Depression symptoms were evaluated using the BDI-II score obtained from most participants in both datasets. Subject data with BDI-II score was used to construct the BDI prediction model. In total, we used 477 participants (367 HCs and 110 MDDs) in the discovery dataset and 439 patients (259 HCs and 180 MDDs) in the validation dataset. This study was carried out in accordance with recommendations of the institutional review boards of the principal investigators' respective institutions (Hiroshima University, Kyoto University, Showa University, University of Tokyo, and Yamaguchi University) with written informed consent from all subjects in accordance with the Declaration of Helsinki. The protocol was approved by the institutional review boards of the principal investigators' respective institutions as listed above. Most data utilized in this study can be downloaded publicly from the DecNef Project Brain Data Repository at https://bicr-resource.atr.jp/srpbsopen/ and https://bicr.atr.jp/dcn/en/download/harmonization/. Data availability statements of each site are described in Supplementary Table 1. Note, although the datasets used in this study are published in our previous study (13), the aims, analyses, results, conclusions, and implications are independent in the current study.

### Preprocessing and Calculation of the Resting State FC Matrix

Rs-fMRI data was preprocessed using FMRIPREP version 1.0.8 (60). The first 10 s of data were discarded to allow for T1 equilibration. We conducted slice-timing correction, realignment, co-registration, distortion correction using a field map, segmentation of T1-weighted structural images, normalization to Montreal Neurological Institute (MNI) space, and spatial smoothing with an isotropic Gaussian kernel of 6 mm full-width at half-maximum. We performed “Fieldmap-less” distortion correction for the validation dataset due to the lack of field map data. For more details on the pipeline, see http://fmriprep.readthedocs.io/en/latest/workflows.html. For data from 6 participants in the validation dataset, co-registration was unsuccessful, and these data were excluded from further analysis.

### Parcellation of Brain Regions

To analyze data using Human Connectome Project (HCP)-style surface-based methods, ciftify toolbox version 2.0.2 was used (61). This allowed us to analyze our data, which lacked T2-weighted images required for HCP pipelines, using an HCP-like surface-based pipeline. We then extracted BOLD signal time courses from Glasser's 379 surface-based parcellations (360 cortical parcellations + 19 subcortical parcellations) as regions of interest (ROIs), considered reliable brain parcellations (36). To facilitate comparisons of our results with previous studies, we identified anatomical names of important ROIs and names of intrinsic brain networks that included ROIs using anatomical automatic labeling (AAL) (62) and Neurosynth (http://neurosynth.org/locations/).

### Physiological Noise Regression

We extracted physiological noise regressors by applying anatomical CompCor (aCompCor) (63) in which principal components were estimated. A mask to exclude signals with cortical origins was obtained by eroding the brain mask and ensuring that it contained subcortical structures only. Five aCompCor components were calculated within the intersection of the subcortical mask and union of the CSF and WM masks calculated in T1-weighted image space after their projection to the native space of functional images in each session. To remove several sources of spurious variance, we used a linear regression with 12 regression parameters as total, such as 6 motion parameters, the average signal over the whole brain, and 5 aCompCor components.

### Temporal Filtering

We applied a temporal bandpass filter to the time series using a first-order Butterworth filter with a pass band between 0.01 and 0.08 Hz to restrict the analysis to low-frequency fluctuations, which are characteristic of rs-fMRI BOLD activity (64).

### Head Motion

We calculated framewise displacement (FD) (65) for each session using Nipype (https://nipype.readthedocs.io/en/latest/). The FD represents head motion between 2 consecutive volumes as a scalar quantity, i.e., the summation of absolute displacements in translation and rotation. We used FD in the subsequent scrubbing procedure. To reduce spurious changes in FCs from head motion, volumes with FD > 0.5 mm were removed, as proposed by Power et al. (65). Using the aforementioned threshold, 6.3% ± 13.5 volumes (mean ± SD) were removed for each rs-fMRI session in all datasets. If the ratio of the excluded volumes after scrubbing exceeded the mean + 3 SD, participants were excluded from the analysis. As a result, 32 participants were removed from all datasets. Thus, we included 683 participants (545 HCs, 138 patients with MDD) in the discovery dataset and 440 participants (259 HCs, 181 patients with MDD) in the validation dataset for further analysis.

### Calculation of FC Matrix

We calculated FC as the temporal correlation of rs-fMRI BOLD signal time courses across 379 ROIs for each participant. There are a number of different candidates to calculate FC, such as the tangent method and partial correlation; however, we used a Pearson's correlation coefficient because it is the most commonly used in previous studies. We calculated Fisher's z-transformed Pearson's correlation coefficients between the preprocessed BOLD signals of each possible pair of ROIs and used to construct 379 × 379 symmetrical connectivity matrices in which each element represents a connection strength between 2 ROIs. In total, 71,631 FC values [(379 × 378)/2] of the lower triangular matrix of the connectivity matrix were used for further analysis.

### Site-Difference Control in FC

A traveling subject harmonization method was used to control for site differences in FC in the discovery dataset. This method enabled us to remove pure site differences (measurement bias) which are estimated from the traveling subject dataset wherein multiple participants travel to multiple imaging sites. We estimated participant factor (***p***), measurement bias (***m***), sampling biases (***s***_hc_, ***s***_mdd_), and psychiatric disorder factor (***d***) by fitting the regression model to the FC values of all participants from the discovery dataset and the traveling subject dataset. For each connectivity, the regression model can be written as follows:

$$\begin{array}{l} Connectivity={{\text{x}}_{\text{m}}}^{T}\text{m}+{{\text{x}}_{{\text{s}}_{\text{h}\text{c}}}}^{T}{\text{s}}_{\text{h}\text{c}}+{{\text{x}}_{{\text{s}}_{\text{mdd}}}}^{T}{\text{s}}_{\text{mdd}}+{{\text{x}}_{\text{d}}}^{T}\text{d}+\\ {{\text{x}}_{\text{p}}}^{T}\text{p}+const+e,\\ such\;that\;\overset{9}{\underset{j}{\sum }}p_{j}=0,\overset{4}{\underset{k}{\sum }}m_{k}=0,\overset{4}{\underset{k}{\sum }}s_{{hc}_{k}}=0,\overset{3}{\underset{k}{\sum }}s_{{mdd}_{k}}\\ \;=0,\;d_{1}\left(HC\right)=0, \end{array}$$

in which *m* represents the measurement bias (4 sites × 1), **s**_*hc*_ represents the sampling bias of HCs (4 sites × 1), **s**_*mdd*_ represents the sampling bias of patients with MDD (3 sites × 1), *d* represents the disorder factor (2 × 1), *p* represents the participant factor (9 traveling subjects × 1), *const* represents the average functional connectivity value across all participants from all sites, and *e* ~ *N*(0, γ^−1^) represents noise. Measurement biases were removed by subtracting estimated measurement biases. Thus, harmonized functional connectivity values were set as follows:

$$\begin{array}{l} Connectivity^{Harmonized}=Connectivity-{{\text{x}}_{\text{m}}}^{T}\hat{\text{m}}, \end{array}$$

in which $\hat{m}$ represents the estimated measurement bias. More detailed information has been described previously (29).

ComBat harmonization method was used (37–40) to control for site differences in FC in the independent validation dataset due to lack of a traveling subject dataset for those imaging sites. Harmonization was performed to correct only for the site difference using information on MDD diagnosis, BDI score, age, sex, and dominant hand as auxiliary variables in ComBat. Notably, compared with the conventional regression method, the ComBat method is a more advanced method to control for site effects (37–40).

### Control of Age Effect

Linear regression was used to further control for age-related effects in FCs by regressing FC values after harmonization. Resulting residuals were an estimate of the FC controlling for age effects.

### BDI Score Regression Model in the Discovery Dataset

We constructed a linear regression model to predict the BDI score using the discovery dataset based on 71,631 FC values. To construct the linear regression model, we applied a machine-learning technique to participants with BDI score in the discovery dataset. Based on our previous study (41), we assumed that symptom factors were not associated with whole brain connectivity, but rather with a specific subset of connections. Therefore, we conducted linear regression analyses using the LASSO method to select the optimal subset of FCs (44). We employed linear regression using the LASSO method as follows:

$$\begin{array}{l} {Predicted\;BDI}_{sub}={\text{w}}^{T}{\text{c}}_{sub}, \end{array}$$

in which **Predicted** **BDI**_**sub**_ represents the BDI score of a participant; **c**_**sub**_ represents an FC vector for a given participant, and *w* represents the weight vector of the linear regression. The weight vector *w* was determined to minimize

$$\begin{array}{l} J\left(\text{w}\right)=-\frac{1}{n_{sub}}\overset{n_{sub}}{\underset{j=1}{\sum }}|{Predicted\;BDI}_{j}-{Observed\;BDI}_{j}|+\lambda ∥\text{w}{∥}_{1}, \end{array}$$

in which $∥\text{w}{∥}_{1}=\overset{N}{\underset{i}{\sum }}|w_{i}|$ and λ represent hyperparameters that control the amount of shrinkage applied to the estimates. To estimate weights and a hyperparameter λ, we conducted a nested cross validation procedure (Figure 2). In this procedure, we first divided the whole discovery dataset into a training set (9 of 10-folds), which were used for training a model and a test set (a fold of 10-folds) for testing the model. We then fitted a model to each fold while tuning a regularization parameter in the inner loop of the nested cross validation, resulting in 10 regression models. For the inner loop, we used the “*lassoglm*” function in MATLAB (R2016b, Mathworks, USA) and set “NumLambda” to 25 and “CV” to 10. In this inner loop, we first calculated a value of λ just large enough such that the only optimal solution is the all-zeroes vector. A total of 25 values of λ were prepared at equal intervals from 0 to λ_max_ and the λ was determined according to the one-standard-error-rule in which we selected the largest λ within the standard deviation of the minimum prediction error (among all λ) (35). We constructed a regression model using the combination of FC values selected in all 10 folds in the training dataset (Figure 2). This caused information leakage across the folds; therefore, the training dataset may be overfitted. This issue meant that it was important to confirm generalization performance by applying this regression model to an independent validation dataset, as described below. Finally, we calculated the mean absolute error (MAE) and Pearson's correlation coefficients between the predicted and measured BDI scores.

### Generalization Performance of the Regression Model

To test the generalizability of the regression model, we applied the trained regression model to a independent validation dataset and considered its output as the predicted BDI score. To test the statistical significance of the BDI regression model, a permutation test was performed. We permuted the BDI scores of the discovery dataset, conducted a 10-fold CV, repeated this permutation procedure 100 times, and calculated the Pearson correlation coefficients and MAE as the performance of each permutation.

### Identification of FCs Linked to Depression Symptoms

Resting-state FC for depression symptoms were examined by extracting the important FCs related to the BDI regression model. In short, the number of times an FC was selected by LASSO was counted during the 10-fold CV. We considered that this FC was important if this number was significantly higher than chance, according to a permutation test. We permuted the BDI scores of the discovery dataset, conducted a 10-fold CV, and repeated this permutation procedure 100 times. We then used the number of counts for each connection selected by the sparse algorithm during 10 CV (max 10 times) as a statistic in every permutation dataset. To control the multiple comparison problem, we set a null distribution as the max distribution of the number of counts over all functional connections and set our statistical significance to a threshold (*p* < 0.05, one-sided). FCs selected ≥ 1 time of 10 times were regarded as relevant to depression symptoms.

## Data Availability Statement

Publicly available datasets were analyzed in this study. This data can be found at: The raw data utilized in this study can be downloaded publicly from the DecNef Project Brain Data Repository at https://bicr-resource.atr.jp/srpbsopen/ and https://bicr.atr.jp/dcn/en/download/harmonization/.

## Ethics Statement

The studies involving human participants were reviewed and approved by The institutional review boards of our respective institutions (Advanced Telecommunications Research Institute International [approval numbers: 13–133, 14–133, 15–133, 16–133, 17–133, and 18–133], Hiroshima University [E-38], Kyoto Prefectural University of Medicine [RBMR-C-1098], Showa University [SWA] [B-2014-019 and UMIN000016134], the University of Tokyo [UTO] Faculty of Medicine [3150], Kyoto University [C809 and R0027], and Yamaguchi University [H23-153 and H25-85]). The patients/participants provided their written informed consent to participate in this study.

## Author Contributions

AY and MK designed the study. TY, NY, AK, NO, TI, RH, HM, NI, MT, GO, HY, KH, KM, ST, KK, NK, HT, and YO recruited participants for the study, collected their clinical and imaging data, and constructed the database. AY performed data preprocessing. AY and OY performed analysis under the supervision of MK and HI. AY, MK, OY, and HI primarily wrote the manuscript. All authors contributed to the article and approved the submitted version.

## Conflict of Interest

MK, NY, RH, HI, NK, and KK are inventors of a patent owned by Advanced Telecommunications Research (ATR) Institute International related to the present work [PCT/JP2014/061543 (WO2014178322)]. MK, NY, RH, NK, and KK are inventors of a patent owned by ATR Institute International related to the present work [PCT/JP2014/061544 (WO2014178323)]. MK and NY are inventors of a patent application submitted by ATR Institute International related to the present work [JP2015-228970]. AY and MK are inventors of a patent application submitted by ATR Institute International related to the present work [JP2018-192842]. The remaining authors declare that the research was conducted in the absence of any commercial or financial relationships that could be construed as a potential conflict of interest.
