# Supplementary material for: Common Brain Networks Between Major Depressive-Disorder Diagnosis and Symptoms of Depression That Are Validated for Independent Cohorts
Source: Front Psychiatry. 2021 Jun 10;12:667881. doi: 10.3389/fpsyt.2021.667881 (PMC8224760; doi:10.3389/fpsyt.2021.667881)
Supplement: Supplementary file 1 [file Data_Sheet_1.pdf]

# 1 Supplementary Material

## 1.1 S1 Text. Higher/lower resolution of regions of interest

We assessed the prediction performance when we used a parcellation scheme other than Glasser's region of interest (ROI). We investigated the effect of higher or lower resolution in the parcellation on prediction performance using Schaefer's ROI (Schaefer et al., 2018) and same procedure in the main text. Since there are 100 to 1,000 ROIs in increments of 100, Schaefer's ROI is suitable for investigating the effect of higher or lower resolution parcellations. We excluded 1,000 ROIs from this analysis because the scanned volume did not cover all 1,000 ROIs. Furthermore, we could not construct a prediction model when we used 100, 200, 400, 600, or 700 ROIs, because no FC was selected by LASSO during the hyperparameter determination. As a result, prediction performances for the independent validation dataset were $r = 0.20$ (300 ROI), $r = 0.13$ (500 ROI), $r = 0.11$ (800 ROI), and $r = 0.07$ (900 ROI), respectively. We found that the prediction performance tends to decrease as the number of ROIs increases. As such, our choice of Glasser's 379 ROIs was adequate.

## 1.2 S2 Text. Brain network marker of MDD diagnosis and Important FCs for MDD diagnosis

We constructed a brain network marker for MDD, which distinguished between HCs and MDD patients, using the discovery dataset based on 71,631 FC values. We used logistic regression with LASSO, a sparse machine learning algorithm, to select the optimal subset of FCs. To estimate the weights of logistic regression and a hyperparameter that determines how many FCs were used, we conducted a nested cross-validation procedure. We first divided the whole discovery dataset into the training set (9 folds out of 10 folds), which was used for training a model, and the test set (1 fold out of 10 folds), which was used for testing the model. To avoid bias due to the difference in the number of patients with MDD and HCs, we used an undersampling method for equalizing the numbers between the MDD and HC groups. Since only a subset of training data was used after undersampling, we repeated the random sampling procedure 10 times (i.e., subsampling). When we performed undersampling and subsampling procedures, we matched the mean age between MDD and HC groups in each subsample. We then fitted a model to each subsample while tuning a regularization parameter in an inner loop of nested cross validation, resulting in 10 classifiers. The mean classifier-output value (diagnostic probability) was considered indicative of the classifier output. Diagnostic probability values of >0.5 were considered indicative of an MDD diagnosis.

## Prediction model of depression symptoms

The classifier distinguished MDD and HC populations with an accuracy of 66% in the discovery dataset. The corresponding AUC was 0.74, indicating acceptable discriminatory ability. We tested the generalizability of the classifier using an independent validation dataset. We created 100 classifiers of MDD (10-fold × 10 subsamples); therefore, we applied all trained classifiers to the independent validation dataset. Next, we averaged the 100 outputs (diagnostic probability) for each participant and considered the participant as a patient with MDD if the averaged diagnostic probability value was >0.5. The classifier distinguished the MDD and HC populations with an accuracy of 66% in the independent validation dataset. If the accuracy for the validation dataset is much smaller than that of the discovery dataset, overfitting is strongly suggested, and the reproducibility of the results is put into doubt. In our case, 66% accuracy for the validation dataset was actually the same as the 66% accuracy for the discovery dataset, so this concern does not apply. The corresponding AUC was 0.74, indicating an acceptable discriminatory ability.

We examined important resting-state FCs for an MDD diagnosis. Briefly, we counted the number of times an FC was selected by LASSO during the 10-fold cross-validation (CV). We considered this FC to be important if this number was significantly higher than the threshold for randomness, according to a permutation test. We permuted the diagnostic labels of the discovery dataset and conducted a 10-fold CV and 10-subsampling procedure, and we repeated this permutation procedure 100 times. We then used the number of counts for each connection selected by the sparse algorithm during 10 subsamplings × 10-fold CV (max 100 times) as a statistic in every permutation dataset. set a null distribution as the max distribution of the number of counts over all FCs and set our statistical significance to a certain threshold (permutation test, $P < 0.05$, 1-sided). S1 Fig shows the spatial distribution of the 25 FCs that were automatically and unbiasedly identified from the data for the reliable classification of MDD and HC by the machine learning algorithms.

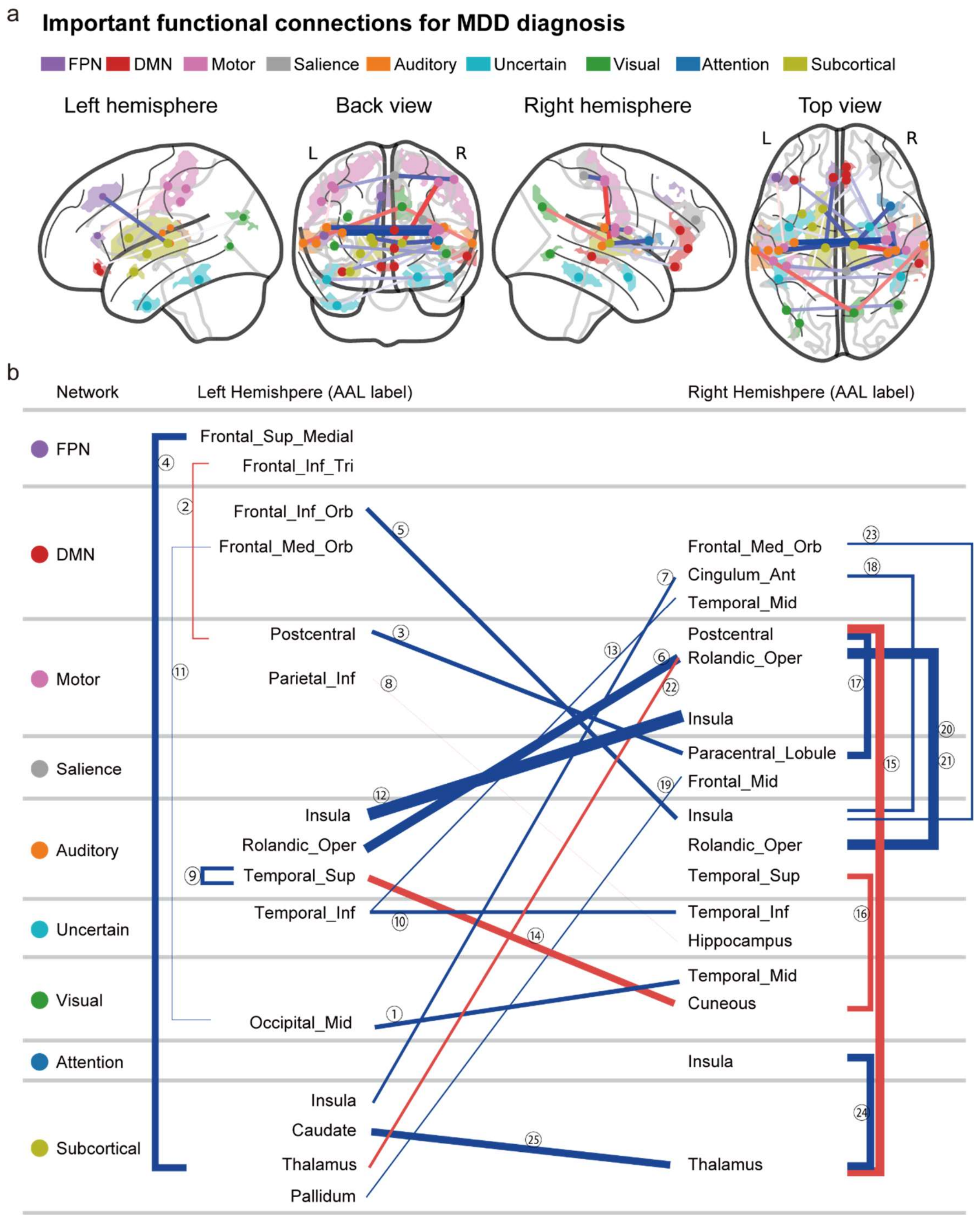


**S1 Fig. Important FCs for MDD diagnosis.** (a) The 25 FCs viewed from left, back, right, and top. Interhemispheric connections are shown in the back and top views only. Regions are color-coded according to the intrinsic network. States of functional connectivity exhibiting smaller (more negative) or greater (more positive) mean correlation indexes in the MDD population than in the HC population are termed under- (blue line) and over-connectivity (red line), respectively. The width of the line represents the effect size of the difference (t-value) in the FC values between MDD and HC groups. (b) Listed here are the laterality and anatomical identification of the ROI as identified by the AAL and associated intrinsic networks related to the 25 FCs. AAL, anatomical automatic labeling; DMN, default mode network; FC, functional connectivity; FPN, fronto-parietal network; HC, healthy control; MDD,

major depressive disorder; ROI, region of interest. (adapted from Yamashita et al. 2020) (Yamashita et al., 2020)

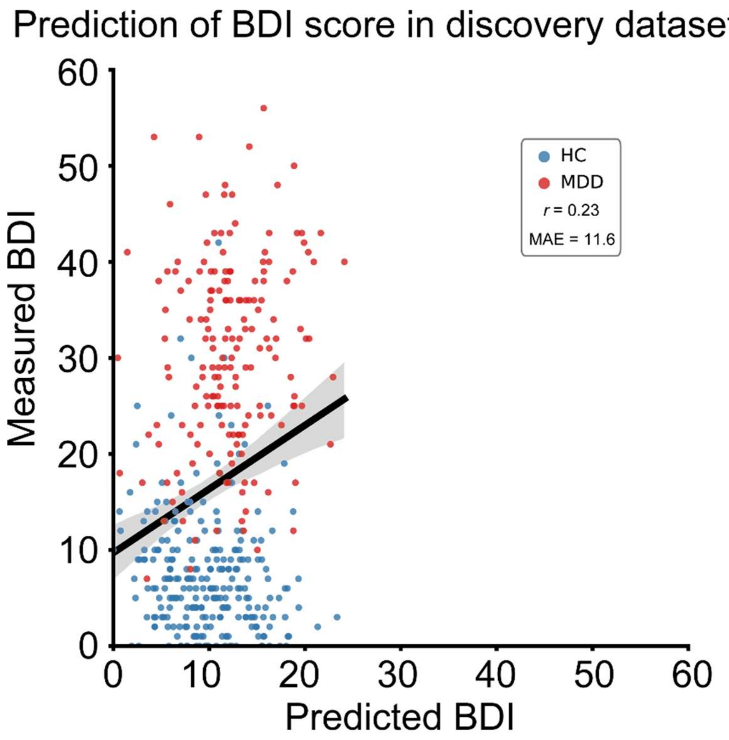


**S2 Fig. BDI regression model performances in the validation datasets using SVR.** Scatter plots of measured and predicted BDI in the independent validation dataset. The solid line indicates the linear regression of the measured BDI from the predicted BDI. The correlation coefficient (*r*) and mean absolute error (MAE) are shown. Each data point represents one participant. BDI: Beck Depression Inventory-II; HC: healthy control; MDD: major depressive disorder.

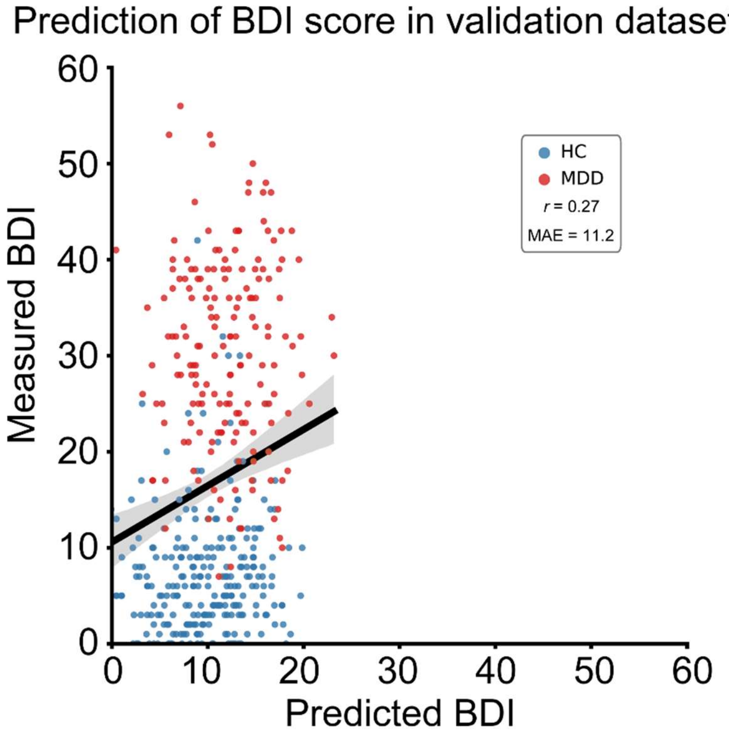


**S3 Fig. BDI regression model performances in the validation datasets using ComBat harmonization.** Scatter plots of measured and predicted BDI in the independent validation dataset. The solid line indicates the linear regression of the measured BDI from the predicted BDI. The correlation coefficient (*r*) and mean absolute error (MAE) are shown. Each data point represents one participant. BDI: Beck Depression Inventory-II; HC: healthy control; MDD: major depressive disorder.

**Supplementary Table 1 | Imaging protocols for resting-state fMRI in both datasets**

| Site | Center of Innovation in Hiroshima University | Kyoto University TimTrio | Showa University | University of Tokyo | Hiroshima Kajikawa Hospital | Hiroshima Rehabilitation Center | Hiroshima University Hospital | Yamaguchi University | Kyoto University Trio |
|---|---|---|---|---|---|---|---|---|---|
| Abbreviation | COI | KUT | SWA | UTO | HKH | HRC | HUH | UYA | KTT |
| MRI scanner | *Siemens Verio* | *Siemens TimTrio* | *Siemens Verio* | *GE MR750w* | *Siemens Spectra* | *GE Signa HDxt* | *GE Signa HDxt* | *Siemens Skyra* | *Siemens Trio* |
| Magnetic field strength | 3.0 T | | | | | | | | |
| Channels per coil | 12 | 32 | 12 | 24 | 12 | 8 | 8 | 20 | 8 |
| Field-of-view (mm) | 212 × 212 | | | | 192 × 192 | 256 × 256 | 256 × 256 | 220 × 220 | 256 × 192 |
| Matrix | 64 × 64 | | | | | | | | 64 × 48 |
| Number of slices | 40 | | | | 38 | 32 | 32 | 34 | 30 |
| Number of volumes | 240 | | | | 107 | 143 | 143 | 200 | 180 |
| In-plane resolution (mm) | 3.3125 × 3.3125 | | | | 3.0 × 3.0 | 4.0 × 4.0 | 4.0 × 4.0 | 3.4 × 3.4 | 4.0 × 4.0 |
| Slice thickness (mm) | 3.2 | | | | 3.0 | 4 | 4.0 | 4.0 | 4.0 |
| Slice gap (mm) | 0.8 | | | | 0 | 0 | 0 | 1.0 | 0 |
| TR (ms) | 2500 | | | | 2,700 | 2,000 | 2,000 | 2,500 | 2,000 |
| TE (ms) | 30 | | | | 31 | 27 | 27 | 30 | 30 |
| Total scan time (min:s) | 10:00 | | | | 5:00 | 4:46 | 5:00 | 8:28 | 6:00 |
| Flip angle (degree) | 80 | | | | 90 | 90 | 90 | 80 | 90 |
| Slice acquisition order | Ascending | | | | Ascending | Ascending (Interleaved) | Ascending (Interleaved) | Ascending | Ascending (Interleaved) |
| Phase encoding | AP | PA | PA | PA | AP | AP | PA | PA | AP |
| Eyes closed/ open/ fixate | Fixate | | | | Fixate | Fixate | Fixate | Closed | Fixate |
| *Type of data availability | 1 | 2 | 2 | 2 | 1 | 1 | 1 | 4 | 2 |

*Type of data availability, 1) freely available without restriction, allowing commercial reuse, 2) freely available, but not allowing commercial reuse, 3) available after registration to our record, but not allowing commercial reuse, 4) available only to our research group

| Supplementary Table 2 \| Clinical characteristics of major depressive disorder patients in the discovery dataset | | | |
|---|---|---|---|
| **Site** | Center of Innovation in Hiroshima University (COI) | Kyoto University (KUT) | University of Tokyo (UTO) |
| ***HAMD17 total (mean±1SD)*** | 15.7 ± 5.1 | 13.1 ± 5.1 | 10.8 ± 6.3 |
| ***Diagnostic criteria*** | MINI | SCID | SCID |
| ***Duration of disease*** *(since the first onset)* | NA | 11.0 ± 5.4 (yr) | 9.0 ± 7.7 (yr) |
| ***Presence of suicide attempt*** | 55 % | 6 % | 18 % |
| ***Psychiatric comorbidities*** | | | |
| ***GAD*** | 3 % | 6 % | 0 % |
| ***OCD*** | 7 % | 6 % | 0 % |
| ***ASD*** | 0 % | 6 % | 0 % |
| ***Panic*** | 0 % | 13 % | 0 % |
| ***Psychiatric medications*** | | | |
| ***Anxiolytic*** | 51 % | 63 % | 77 % |
| ***Antipsychotic*** | 24 % | 31 % | 32 % |
| ***Mood stabilizer*** | 6 % | 6 % | 45 % |
| ***Antidepressant*** | 90 % | 94 % | 69 % |
| ***Subtype of major depressive disorder*** | | | |
| ***Melancholic*** | 64 % | NA | 39 % |
| ***Treatment resistance*** | NA* | 100% | NA |

*Not applicable because of early treatment data. MINI: Mini International Neuropsyciatric Interview, SCID: Structured Clinical Interview for DSM-IV, NA: Not applicable, HAMD: Hamilton Depression Rating Scale.

**Supplementary Table 3 | All functional connections related to depression symptoms**

| ID | ROI1 | | | ROI2 | | | *r*-value with BDI (Discovery) | *r*-value with BDI (Validation) |
|---|---|---|---|---|---|---|---|---|
| | Glasser | AAL label | Network | Glasser | AAL label | Network | | |
| **1** | L.3b | Postcentral_L | Motor | R.Thalamus | Thalamus_R | Subcortical | 0.279 | 0.201 |
| **2** | L.PIT | Fusiform_L | Visual | R.PIT | Occipital_Inf_R | Visual | -0.190 | -0.079 |
| **3** | L.A1 | Rolandic_Oper_L | Auditory | R.8Av | Frontal_Mid_R | FPN | 0.166 | -0.010 |
| **4** | L.7Pm | Precuneus_L | MR | R.TE1m | Temporal_Mid_R | Uncertain | 0.179 | 0.031 |
| **5** | L.9m | Frontal_Sup_Medial_L | DMN | R.9m | Frontal_Sup_Medial_R | DMN | -0.208 | -0.086 |
| **6** | L.44 | Frontal_Inf_Oper_L | FPN | L.TE1p | Temporal_Mid_L | DMN | -0.214 | -0.078 |
| **7** | L.p9-46v | Frontal_Inf_Tri_L | FPN | L.H | Hippocampus_L | Uncertain | 0.165 | 0.059 |
| **8** | L.47s | Frontal_Inf_Orb_L | Uncertain | R.FOP2 | Rolandic_Oper_R | Motor | -0.170 | 0.016 |
| **9** | L.PBelt | Temporal_Sup_L | Auditory | L.A4 | Temporal_Sup_L | Auditory | -0.231 | -0.086 |
| **10** | L.TGd | Temporal_Pole_Mid_L | DMN | R.STSvp | Temporal_Mid_R | DMN | -0.210 | 0.011 |
| **11** | L.PI | Temporal_Sup_L | Attention | R.TE1m | Temporal_Mid_R | Uncertain | -0.203 | 0.004 |
| **12** | R.MIP | Occipital_Sup_R | Attention | R.LBelt | Temporal_Sup_R | Auditory | 0.182 | 0.085 |
| **13** | R.a24 | Cingulum_Ant_R | DMN | R.52 | Insula_R | Auditory | -0.236 | -0.070 |
| **14** | R.52 | Insula_R | Auditory | R.s32 | Frontal_Med_Orb_R | DMN | -0.240 | -0.030 |
| **15** | R.A5 | Temporal_Sup_R | Auditory | R.A4 | Temporal_Sup_R | Auditory | -0.200 | -0.064 |
| **16** | L.Putamen | Putamen_L | Subcortical | R.Putamen | Putamen_R | Subcortical | -0.250 | -0.163 |

ROI labels were determined by referring to AAL and Neurosynth (http://neurosynth.org/locations/). Shaded rows indicate overlap connectivity between depression symptoms related connectivity and major depressive disorder related connectivity. DMN: Default mode network; FPN: Fronto-parietal task control; MR: Memory retrieval

## References


Schaefer, A., Kong, R., Gordon, E.M., Laumann, T.O., Zuo, X.N., Holmes, A.J., Eickhoff, S.B., and Yeo, B.T.T. (2018). Local-Global Parcellation of the Human Cerebral Cortex from Intrinsic Functional Connectivity MRI. *Cereb Cortex* 28**,** 3095-3114.

Yamashita, A., Sakai, Y., Yamada, T., Yahata, N., Kunimatsu, A., Okada, N., Itahashi, T., Hashimoto, R., Mizuta, H., Ichikawa, N., Takamura, M., Okada, G., Yamagata, H., Harada, K., Matsuo, K., Tanaka, S.C., Kawato, M., Kasai, K., Kato, N., Takahashi, H., Okamoto, Y., Yamashita, O., and Imamizu, H. (2020). Generalizable brain network markers of major depressive disorder across multiple imaging sites. *PLOS Biology* 18**,** e3000966.
